# Molecular Analyses of Petroleum Hydrocarbon Change and Transformation during Petroleum Weathering by Multiple Techniques

**DOI:** 10.1021/acsomega.1c02846

**Published:** 2021-08-31

**Authors:** Yazhuo Li, Hui Wang, Zhengqing Cai, Jibiao Zhang, Jie Fu

**Affiliations:** † Department of Environmental Science and Engineering, Fudan University, Shanghai 200433, China; ‡ School of Environmental Science and Engineering, Huazhong University of Science and Technology, Wuhan 430074, China; § SINOPEC Research Institute of Petroleum Processing, Beijing 100083, China; ∥ National Engineering Laboratory for High-Concentration Refractory Organic Wastewater Treatment Technologies, East China University of Science and Technology, Shanghai 200237, China

## Abstract

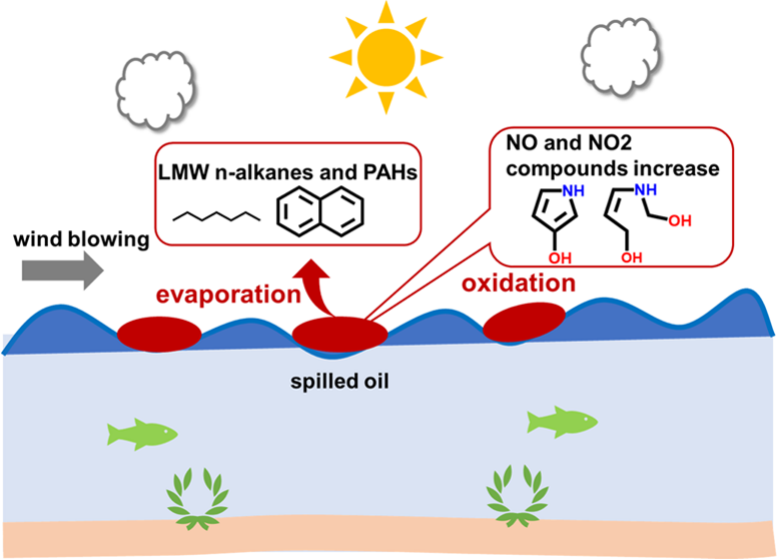

Various analytical techniques are used to study the weathering process of four crude oils, i.e., Iranian light crude oil, Daqing crude oil, Shengli crude oil, and Tahe crude oil. The molecular composition and structural information of *n*-alkanes, polycyclic aromatic hydrocarbons (PAHs), and heteroatom compounds were characterized by gas chromatography-flame ionization detector (GC-FID), gas chromatography-mass spectrometry (GC-MS), and Fourier transform ion cyclotron resonance mass spectrometry (FT-ICR MS), respectively. The results showed that the weathering loss of *n*-alkanes was related to the molecular weight, and the low-molecular-weight (LMW) *n*-alkanes were more volatile. The loss degree of LMW naphthalene and alkylation homologues in PAHs was also higher. With the increase in the alkylation degree, the weathering resistance ability of PAHs was enhanced. In the negative-ion ESI FT-ICR MS mode, a total of 16 classes of compounds were detected for neutral nitrogen compounds and acidic compounds in the four crude oils. With the increase in weathering time, the relative abundances of NO, NO2, and O3S compounds gradually increased. In particular, the NO and NO2 compounds with different condensation degrees increased significantly. These results indicated that in addition to the volatilization of hydrocarbon compounds, nitrogen compounds were also oxidized to a certain extent during the weathering process. The provided information would enrich the understanding of the short-term weathering process of petroleum hydrocarbons.

## Introduction

With the development of offshore oil exploration and marine transportation, crude oil leakage has aroused global concern and become an important source of marine environmental pollution.^1^ Marine petroleum pollution refers to the entry of petroleum and its byproducts into the marine environment during the process of mining, refining, storage, and use.^2^ Especially, some major marine accidents have resulted in large amounts of oil spills. For example, about 4.9 million barrels of crude oil were released into the Gulf of Mexico due to the explosion of the Deepwater Horizon (DWH) oil platform in the spring of 2010, making it the largest oil spill in the history of the United States.^3^ The petroleum components entering the ocean undergo a series of physical, chemical, and biological weathering processes, including adsorption, dissolution, volatilization, chemical reaction, and biodegradation.^4^ Oil spills have significantly changed the marine biogeochemical cycle, posing a great threat to the marine ecosystem and human health,^5,6^ which has become the research focus of marine chemistry and biology in recent years.

Spreading, evaporation, dispersion/diffusion, emulsification, and dissolution are the most crucial oil weathering processes at the early stages of the oil spill, while photooxidation, biodegradation, and sedimentation act in the longer term.^7^ The weathering of an oil spill in the marine environment is largely determined by both the properties of leaked oil and the environmental conditions (wave, winds, currents, solar radiation, etc.). Evaporation takes place when the volatile elements of the oil diffuse from the oil and enter the gaseous stage, while the heavier components of oil remain. Evaporation removes most of the volatile fractions of oil from the atmosphere within a short time, leading to the reduction in oil toxicity in the marine environment.^8^ On the contrary, the increased oil viscosity after evaporation could lead to severe physical and chemical effects on the marine environment.^7^ Many researchers have attempted to estimate the oil evaporation rates by treating the oil as a uniform element.^9,10^ However, oil is actually a complicated mixture of a large number of different types of chemical compounds. To accurately estimate evaporation, it is vital to differentiate among the various chemical groups. In addition, during the evaporation process, oxidation also occurs due to the wind of air. Unfortunately, the information on the transformation of petroleum hydrocarbons during the short-term evaporation weathering process has been kept unexplored.

For the characterization of oil components in the weathering studies, gas chromatography-flame ionization detector (GC-FID) and gas chromatography-mass spectrometry (GC-MS) techniques are usually employed.^11,12^ However, due to the complexity of petroleum components, which contain C, H, and other heteroatoms, including N, O, and S, traditional analytical methods cannot fully identify and infer the molecular composition. In recent years, electrospray ionization (ESI) coupled to high-field Fourier transform ion cyclotron resonance mass spectrometry (FT-ICR MS) has been widely used to analyze the composition of heteroatomic compounds in crude oil.^13−15^ FT-ICR MS is a mass spectrometer having high quality, accuracy, resolution, and can completely separate complex mass spectral peaks of petroleum samples.^16^ Moreover, the exact molecular mass corresponding to the mass spectrum peak can be calculated to determine the polar molecular structures containing C, H, O, N, S, and other elements.^17^ Therefore, more information on the molecular transformation of oil components during the weathering process would be explored by FT-ICR MS analysis.

In this paper, the evaporation weathering of four crude oils, including Iranian light (IL) crude oil, Daqing (DQ) crude oil, Shengli (SL) crude oil, and Tahe (TH) crude oil, was simulated by purging with air. The changes in basic compositions of *n*-alkanes and polycyclic aromatic hydrocarbons (PAHs) during the weathering process were characterized using GC-FID and GC-MS. The transformation of heteroatomic compounds was analyzed using negative-ion ESI FT-ICR MS. This study can deepen the understanding of the change and transformation of different petroleum components during the short-term weathering process.

## Results and Discussion

### Mass Loss of Crude Oil during the Weathering Process

**Figure 1:**
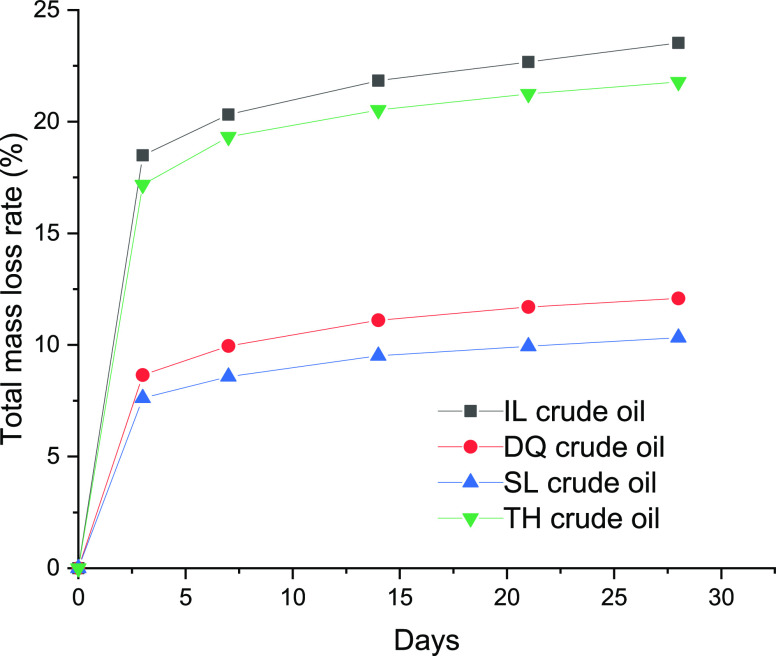
Mass loss rate of different types of crude oils versus weathering time.

Figure 1 shows the mass loss rate of the four crude oils during the weathering process. The loss degree of the oil quality was significant in the first 3 days. Thereafter, the mass loss rates of crude oils gradually increased with the increase in weathering time. Compared with different crude oils, the overall mass loss rates of SL and DQ crude oils were lower than those of IL and TH crude oils. The mass loss rates of SL and DQ crude oils on the 3rd day were only about 10%, while the mass loss rates of IL and TH crude oils reached up to 18%, indicating a more serious weathering degree. Based on the changing trend in the mass loss rate, the crude oils of weathering for 0, 3, and 28 days were selected for subsequent composition analyses. The weathering process is highly impacted by the boiling-point distribution of the original crude samples. The boiling-point distribution before and after weathering is shown in Table S1. It shows a shift in the percent of different boiling-point fraction ranges of the oils on weathering. Generally, the lowest fraction range disappeared and the initial boiling point increased after the weathering process.

### Changes of *n*-Alkane Components during the Weathering Process

**Figure 2:**
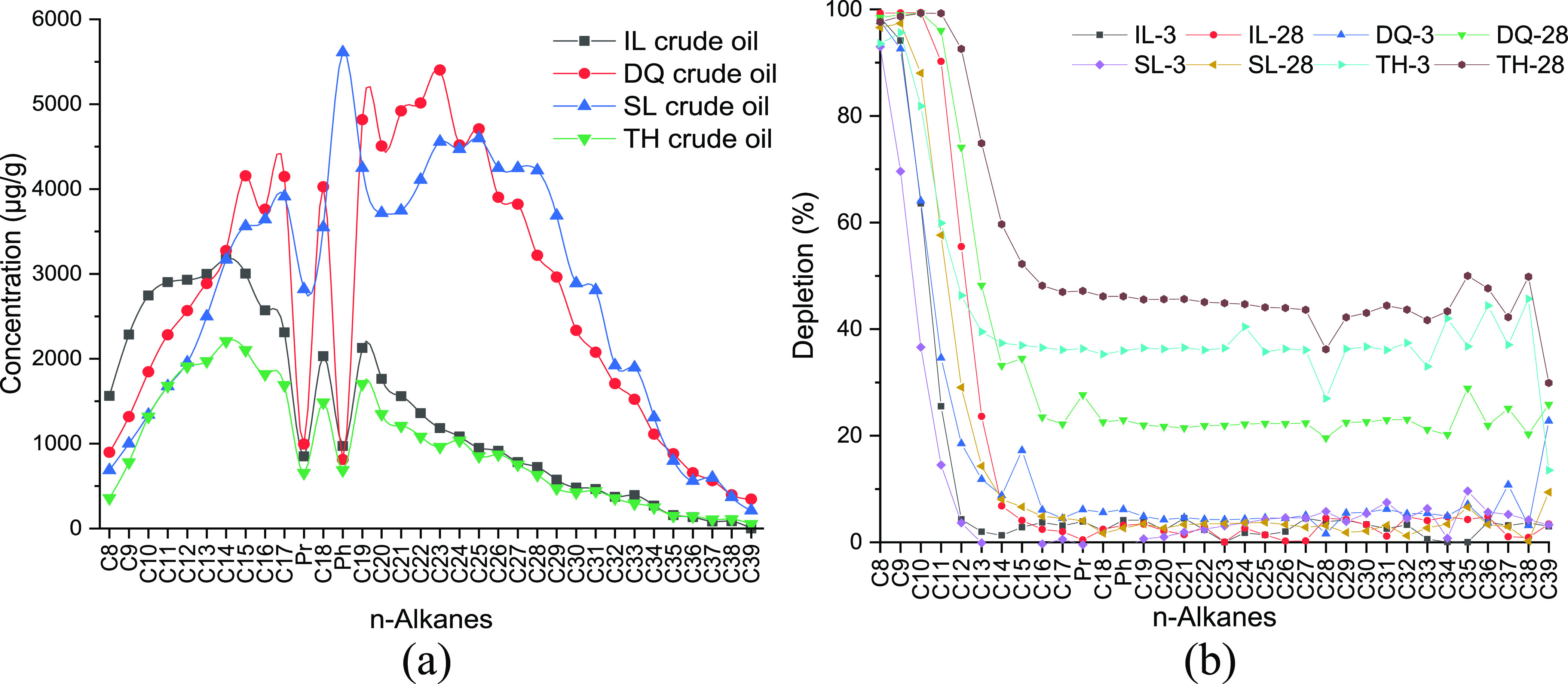
Contents of *n*-alkane components in initial crude oils (a) and depletion of *n*-alkane components in crude oils after weathering for 3 and 28 days (b).

The *n*-alkane components of four crude oil samples were analyzed by GC-FID. The gas chromatograms of *n*-alkanes in the crude oil samples are provided in Figure S1, Supporting Information (SI). As shown in Figure 2a, the distribution range of *n*-alkanes in initial crude oils was C8–C39. The distribution patterns of IL and TH crude oils are very similar. The low-carbon-number *n*-alkane components were both concentrated in C9–C17; however, the *n*-alkane content of IL crude oil was higher than that of TH crude oil before C24. The dominant *n*-alkanes in DQ and SL crude oils were mainly distributed in the range of C15–C30, and the contents were up to 5401.98 μg/g, which are significantly different from those in IL and TH crude oils.

The weathering degree of crude oil was determined based upon the mass loss relative to the conservative internal marker within the oil, viz., 17α(H),21β(H)-hopane (hopane), which has been proven recalcitrant to biodegradation and photooxidation.^18,19^ The depletion of any given fraction in the oils was estimated using the following formula
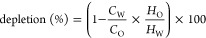
1where *C*_O_ and *C*_W_ are the concentrations of the target compound in the raw oil and weathered oil, respectively, and *H*_O_ and *H*_W_ are the concentrations of hopane in the raw oil and weathered oil.

Figure 2b shows the depletion of *n*-alkanes in the four crude oils after weathering for 3 and 28 days. It can be seen that the weathering loss degree of low-carbon-number *n*-alkanes was relatively large and even close to 100%, which was mainly due to the higher vapor pressure of low-carbon-number *n*-alkanes.^20^ As the carbon number increased, the weathering loss rate gradually decreased and the weathering loss tended to be flat after C16. For the same crude oil, the weathering loss rate increased with the increase in time, while the weathering degree of *n*-alkanes varied from different crude oils.

For IL crude oil, after 3 days of weathering, C8 and C9 were almost completely weathered, C10 and C11 were partially weathered, and C12 was slightly weathered. After 28 days, C10 and C11 were also almost completely weathered, C12 and C13 were partially weathered, and C14 was slightly weathered. The degree of weathering increased significantly, and C15–C39 were basically not weathered. The weathering process of SL crude oil was similar with that of IL crude oil. For DQ crude oil, after 3 days of weathering, C8 and C9 were basically completely weathered, C10–C12 were partially weathered, and C13–C38 were slightly weathered with a weathering rate of only about 5%. After 28 days, the weathering rate of C13–C38 was increased up to 20%. For TH crude oil, *n*-alkanes were greatly affected by weathering, and the weathering rate of C13–C38 reached 35% on the 3rd day and 45% on the 28th day.

### Changes of PAH Components during the Weathering Process

**Figure 3:**
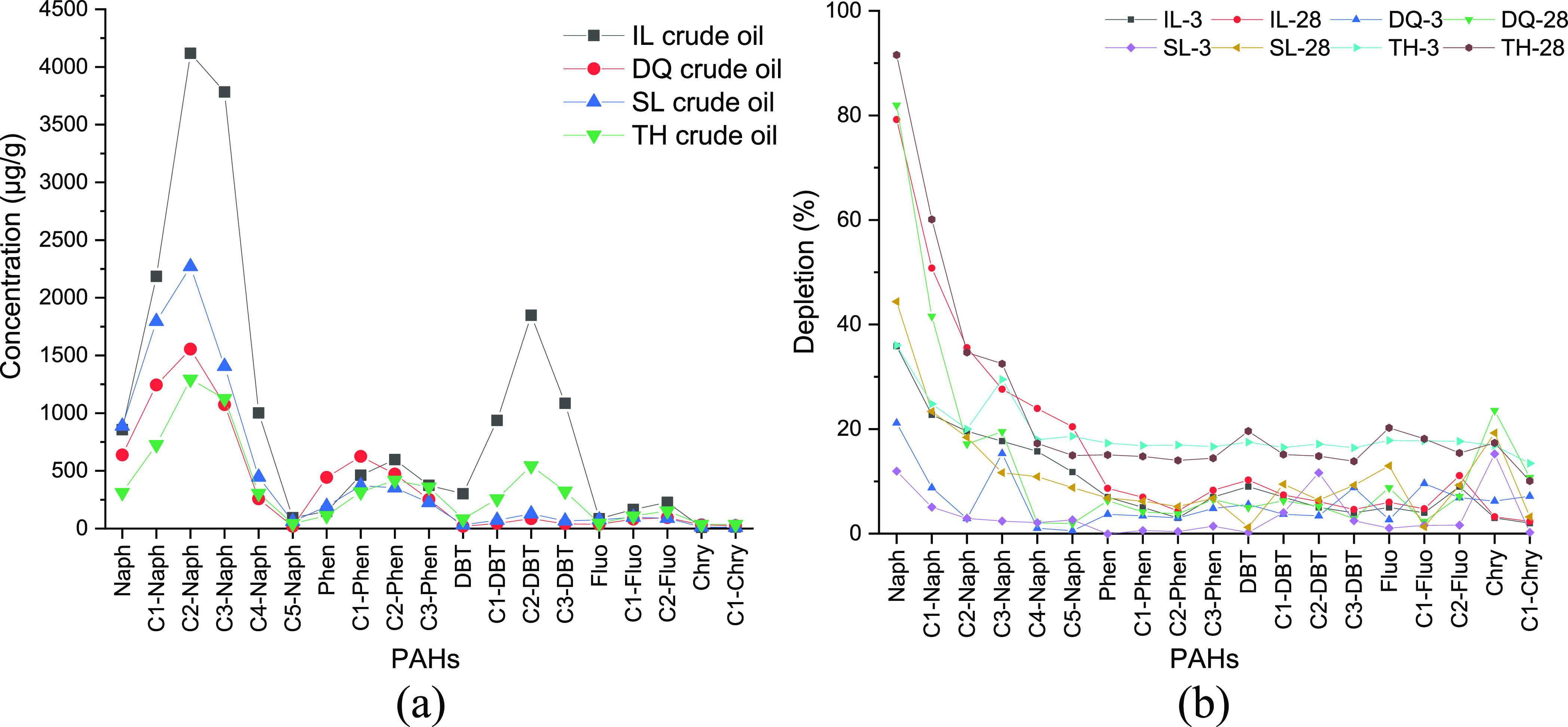
Contents of PAH components in initial crude oils (a) and depletion of PAH components in crude oils after weathering for 3 and 28 days (b).

The PAH components of four crude oil samples were analyzed by GC-MS. The gas chromatograms of PAHs in the crude oil samples are provided in Figure S2, SI. Figure 3a depicts the distribution of PAHs and their alkylated homologues in initial crude oils, including naphthalene (Naph), phenanthrene (Phen), dibenzothiophene (DBT), fluorene (Fluo), chrysene (Chry), and their alkylated compounds.^21,22^ Among PAHs, naphthalene series compounds had the highest content, up to 4000 μg/g, and the degree of alkylation was the highest, containing five carbon-substituted components, followed by Phen and DBT, which contained up to three carbon-substituted groups. The contents of Fluo and Chry compounds were less. Compared with four crude oils, the content of PAHs in IL crude oil was higher, and the content of DBT compounds was 1850 μg/g, much higher than that of Phen compounds of 597 μg/g. Among the alkylation series compounds of PAHs, the distribution trend in crude oils followed the order of C0 < C1 < C2 > C3 > C4 > C5, indicating that PAHs in petroleum were mainly composed of two carbon-substituted components.^23^

Figure 3b shows the depletion of PAH components in the four crude oils after weathering for 3 and 28 days. It can be seen that among the five series PAHs, the depletion of low-molecular-weight (LMW) Naph and alkylated homologues was high and greatly affected by weathering time. For example, the depletion of Naph compounds in DQ crude oil was 20% on the 3rd day and increased to 80% on the 28th day. For IL and SL crude oils, the weathering rate of Naph compounds decreased and weathering resistance increased with the increase in the alkylation degree. However, for DQ and TH crude oils, the loss of C3-Naph on the 3rd day exceeded that of other alkylated Naph. The weathering depletions of Phen and DBT were basically the same, and not affected by the alkylation degree and weathering time. The concentrations of Fluo and Chry in the four crude oils were low, and therefore the real weathering situation was difficult to reflect.

### ESI FT-ICR MS Spectra of Crude Oils during the Weathering Process

Figure S3, SI shows the negative-ion ESI FT-ICR MS mass spectra of crude oil samples during the weathering process. It can be seen that the relative molecular-weight distribution of IL and TH crude oils ranged between 200 and 500 Da, and the mass center was around 350 Da. The mass distribution of DQ and SL crude oils was wide, mainly distributed from 200 to 600 Da, and the mass center was around 450 Da, indicating that the molecular weight of polar components in the two crude oils was high.^24^ There are no obvious changes in the spectra of crude oils before and after weathering, while the identified peaks reflected the complexity of polar compounds. For instance, after 3 and 28 days of weathering, the peak number of IL crude oil showed a gradually increasing trend. For DQ and SL crude oils, the peak number first increased and then decreased, while for TH crude oil, the peak number first decreased and then increased.

### Types and Distribution Characteristics of Heteroatomic Compounds

**Figure 4:**
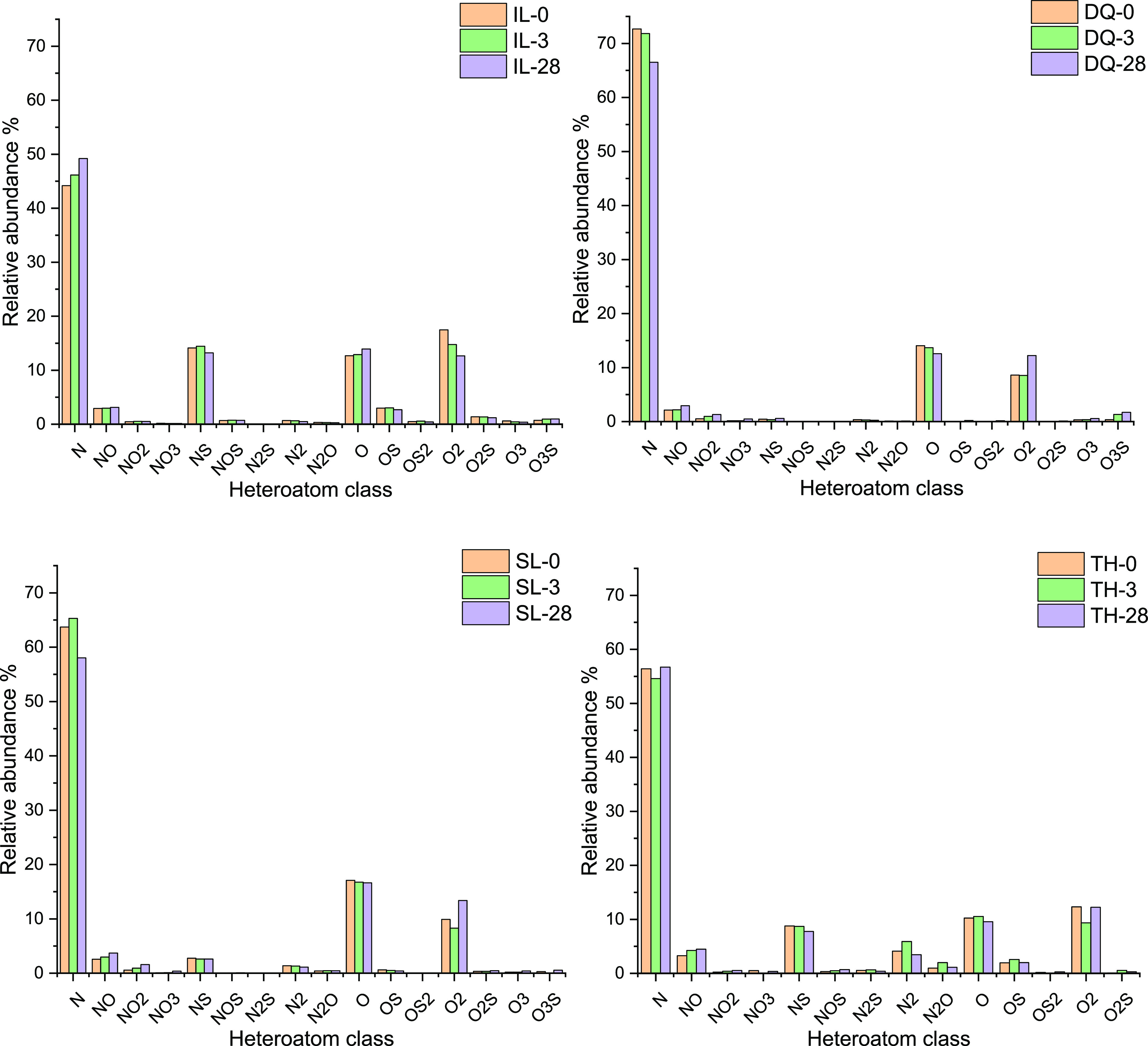
Types and relative abundances of heteroatomic compounds in crude oil samples.

The compounds identified by ESI FT-ICR MS were mainly acidic oxygen-containing compounds and nonbasic nitrogen-containing compounds.^25^ To describe the molecular composition differences between different crude oils, compounds were identified based on the precise molecular weight of mass spectrum peaks and classified based on heteroatomic types.^26^ The relative abundances of heteroatomic compounds are shown in Figure 4.

A total of 16 classes of compounds were detected, including N1, O1, O2, N1S1, N1O1, N2, and O1S1. N1, O1, and O2 were ubiquitous in all crude oil samples and their relative abundances were high.^27^ The relative abundance of the N1 compounds accounted for 45–75%, mainly consisted of pyrrole compounds,^26^ which was far higher than other heteroatomic compounds. O1 and O2 took the second place, and each of them occupied about 10–17%. The types and relative abundances of heteroatomic compounds between different crude oils were obviously different. For IL and TH crude oils, the contents of N1S1 compounds were also high and accounted for 15 and 10%, respectively, while the contents of other compounds were less than 5%. With the increase in weathering time, the relative abundances of heteroatomic compounds have undergone varying degrees of increase or decrease, especially the nitrogenous compounds, N, N2, NS, NO, and NO2.

### Composition and Distribution of N1, O1, and O2 Compounds in Initial Crude Oils

Petroleum is one of the most complex compounds in the natural environment, covering almost all compounds composed of oxygen, hydrogen, carbon, nitrogen, sulfur, and other elements. Compounds with the same number of heteroatoms were grouped by high-resolution mass spectrometry, and those with the same number of the double-bond equivalent (DBE), i.e., the sum of double bonds and rings, were divided into groups. The DBE could indicate the condensation degree of the compounds and is used to infer the molecular structure of the compound.^27^

**Figure 5:**
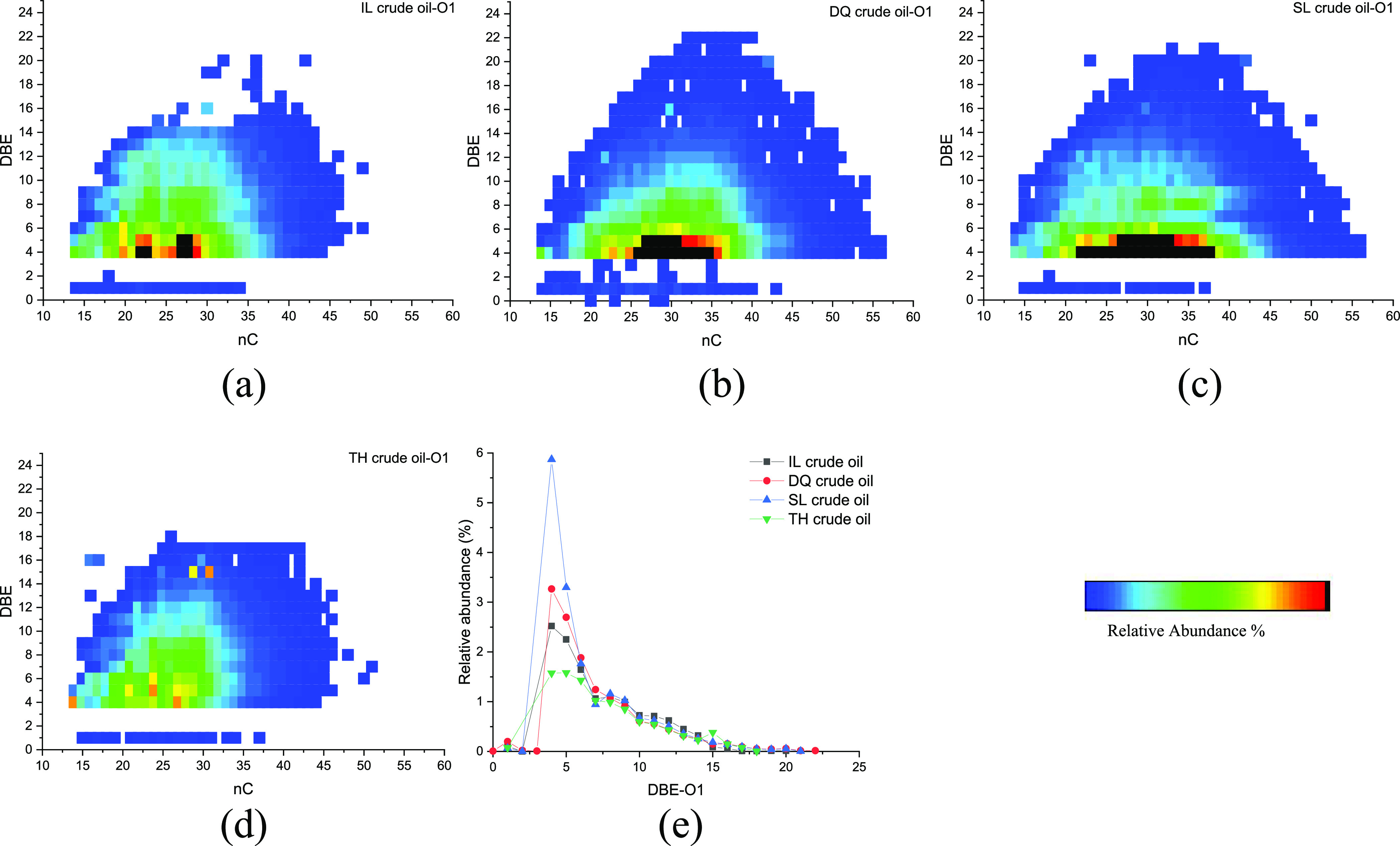
Relation diagram of the carbon number and DBE of O1 compounds (a–d) and the distribution diagram of DBE (e) in initial crude oils.

Figure 5 shows the relation of the carbon number and DBE of O1 compounds and distribution of DBE in the four initial crude oils. O1 species detected under a negative-ion ESI mode contain a hydroxyl moiety,^28^ and the carbon number ranges from 15 to 55. For IL and TH crude oils, the carbon number was mainly distributed in the range of 15–35, while for DQ and SL crude oils, the carbon number was mainly distributed in the range of 20–40. The DBEs ranged from 1 to 22, and compounds with a DBE of 4 were the most abundant O1 species, which were most likely alkyl phenols.^29^ The O1 species with DBEs ≥5 could be assigned to phenols with additional naphthenic or aromatic rings.^17,30^

**Figure 6:**
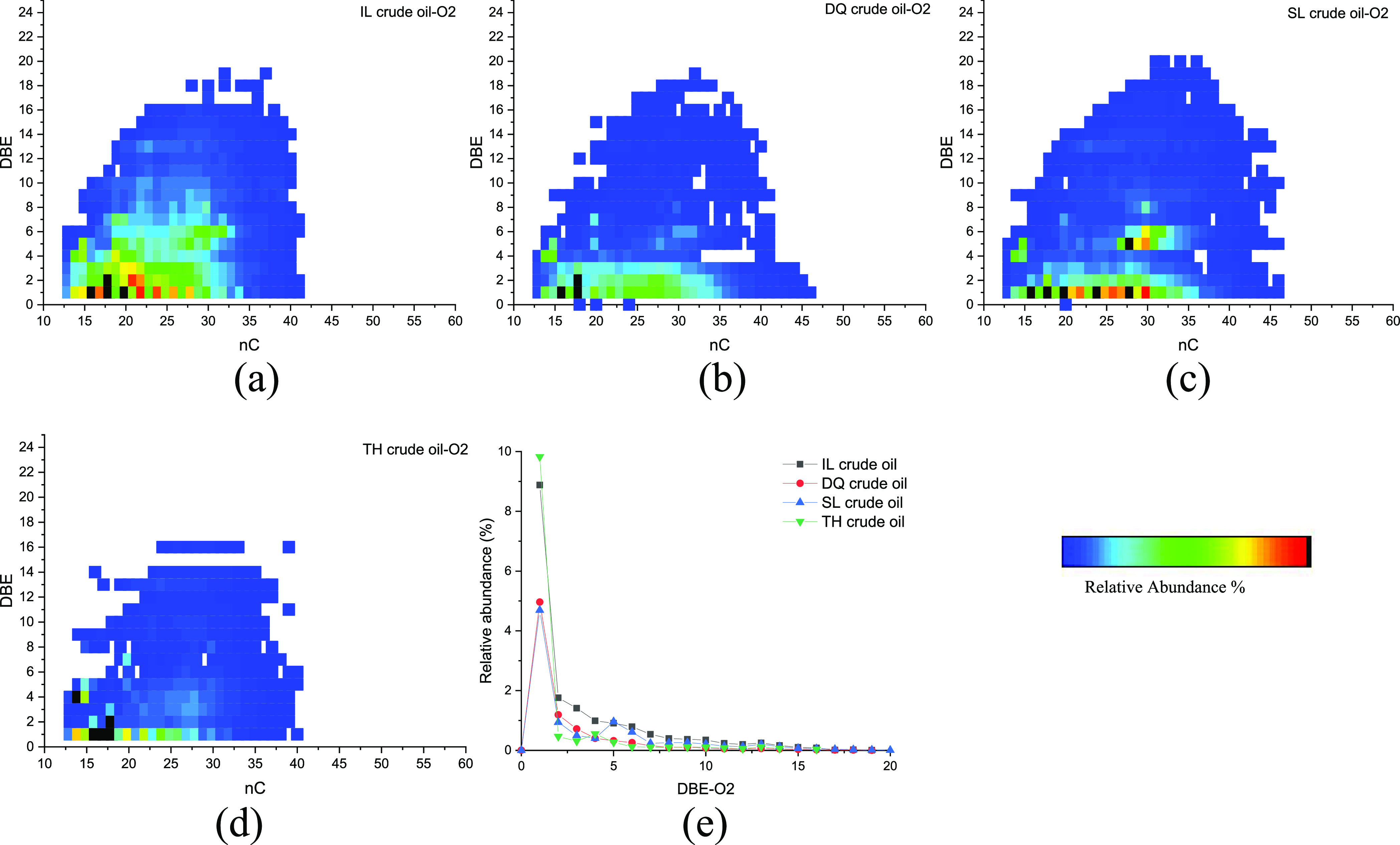
Relation diagram of the carbon number and DBE of O2 compounds (a–d) and the distribution diagram of DBE (e) in initial crude oils.

Figure 6 shows the relation of the carbon number and DBE of O2 compounds and distribution of DBE in the four initial crude oils. O2 species generally correspond to carboxylic acids.^31^ The carbon number of O2 compounds in the four crude oils ranged in 13–45 and was concentrated in 15–30. The DBE ranged from 1 to 20. The compounds with DBE of 1 were the most abundant O2 species, accounting for 10%, which were likely to be fatty acids.^32^ The O2 species with DBEs ≥2 were assigned to naphthenic/aromatic structures containing a carboxyl moiety.^33^ For instance, the O2 species with a DBE range of 2–6 could be naphthenic acids containing one to five naphthenic rings.^34^

**Figure 7:**
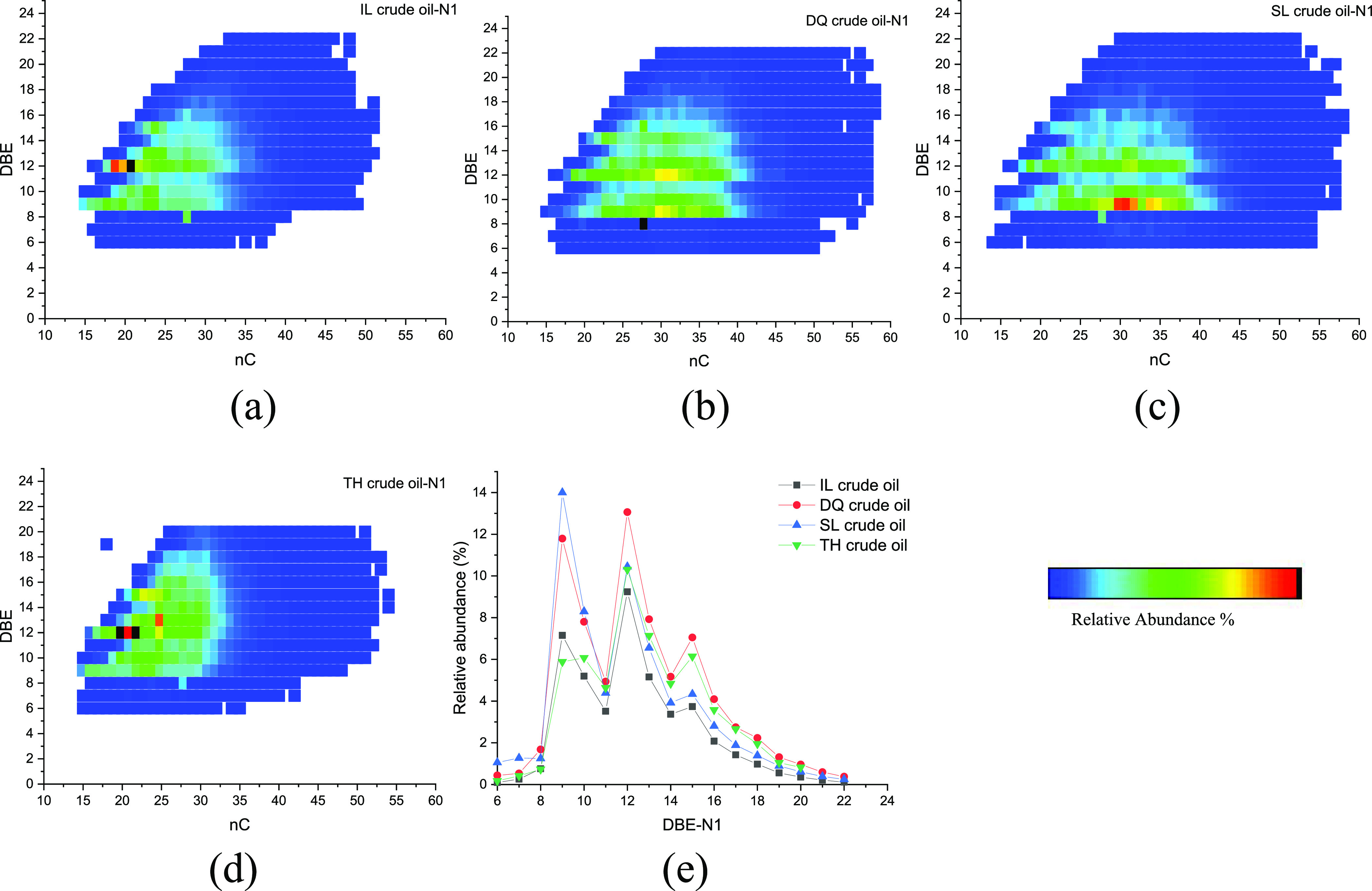
Relation diagram of the carbon number and DBE of N1 compounds (a–d) and the distribution diagram of DBE (e) in initial crude oils.

Figure 7 shows the relation of the carbon number and DBE of N1 compounds and distribution of DBE in the four initial crude oils. N1 species were assigned to neutral nitrogen compounds having a pyrrolic structure (i.e., a nitrogen atom in a five-membered ring).^35^ The detected carbon number of N1 species ranged from 15 to 55. For IL and TH crude oils, the carbon number was mainly distributed in the range of 20–30, while for DQ and SL crude oils, the carbon number was mainly distributed in the range of 20–40. The DBE ranged from 6 to 22, and was concentrated in 9–16, indicating a high unsaturation degree of the compounds.^13^ The abundant DBEs were 9, 12, and 15, responding to carbazole, benzocarbazole, and dibenzocarbazole compounds, respectively.^26,36^

### Changes of Polar Compounds during the Weathering Process

**Figure 8:**
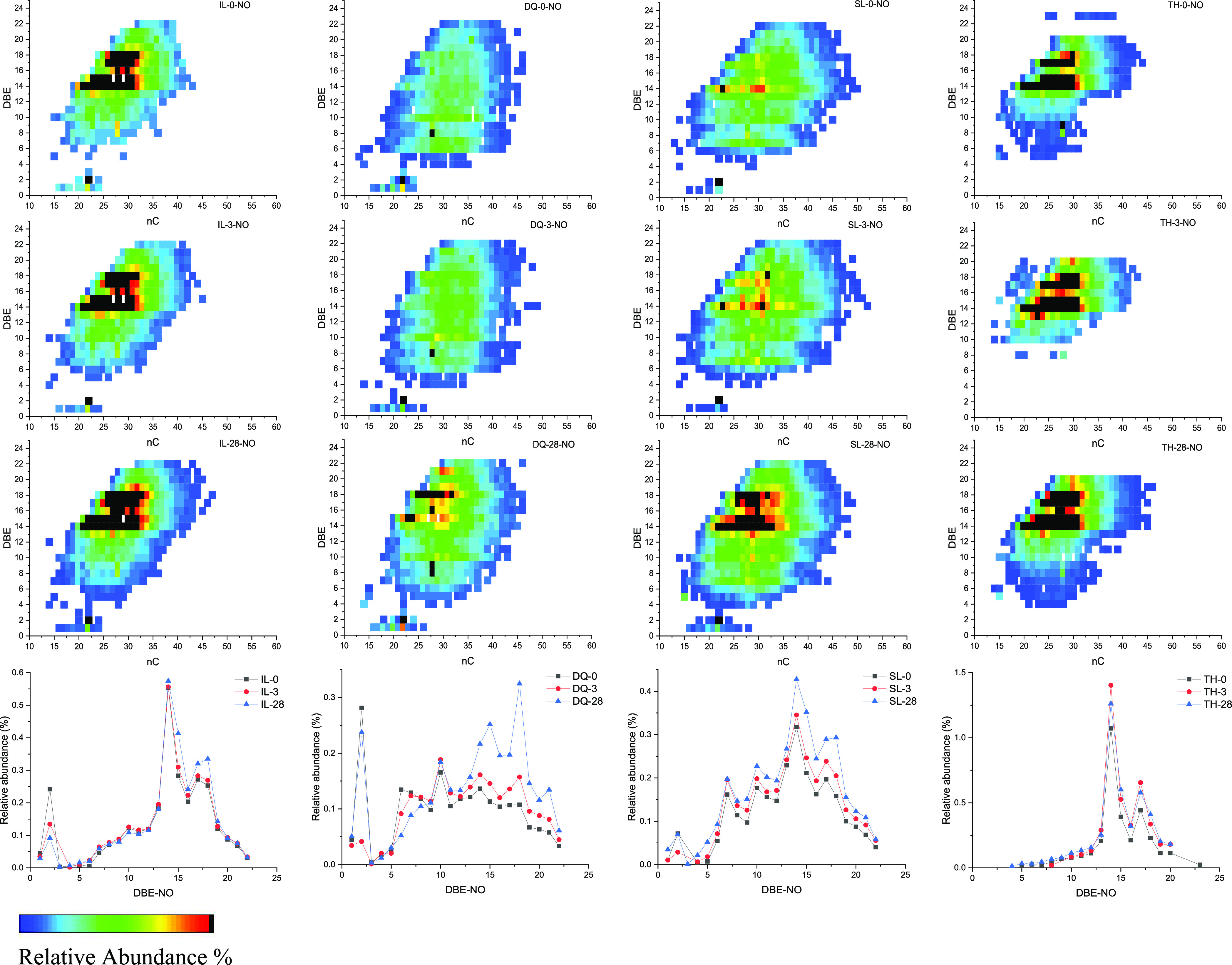
Relation diagram of the carbon number and DBE of NO compounds and changes in the relative abundance of DBE during the weathering process.

**Figure 9:**
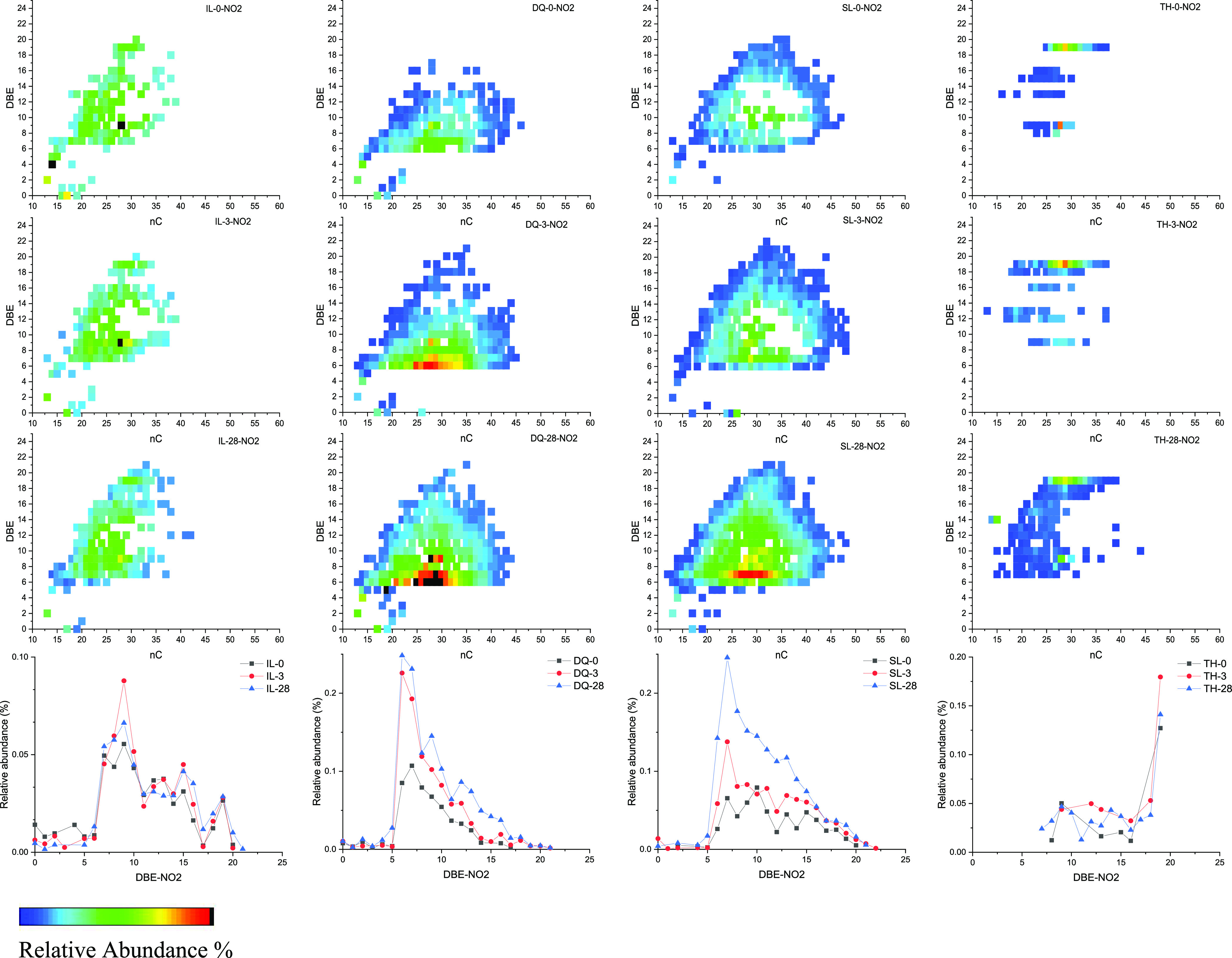
Relation diagram of the carbon number and DBE of NO2 compounds and changes in the relative abundance of DBE during the weathering process.

Figures 8 and 9 show the relation of the carbon number and DBE of NO and NO2 compounds and the changes of the relative abundance of DBE during the weathering process. It can be seen that with the increase in weathering time, the NO and NO2 compounds with different condensation degrees increased significantly to a certain extent, especially for DQ and SL crude oils. For example, the NO2 compounds with DBE = 8–14 and nC = 30–40 in SL crude oil significantly appeared, indicating that there were other nitrogenous compounds were transformed into them. Moreover, the DBE was distributed more widely ranging from 6 to 20 in Tahe oil after weathering for 28 days. These results indicated that in addition to the volatilization of hydrocarbon compounds, nitrogen compounds also underwent oxidation to a certain extent during the weathering process.^13^

### Van Krevelen Diagrams for All Classes Containing Oxygen in Crude Oils

**Figure 10:**
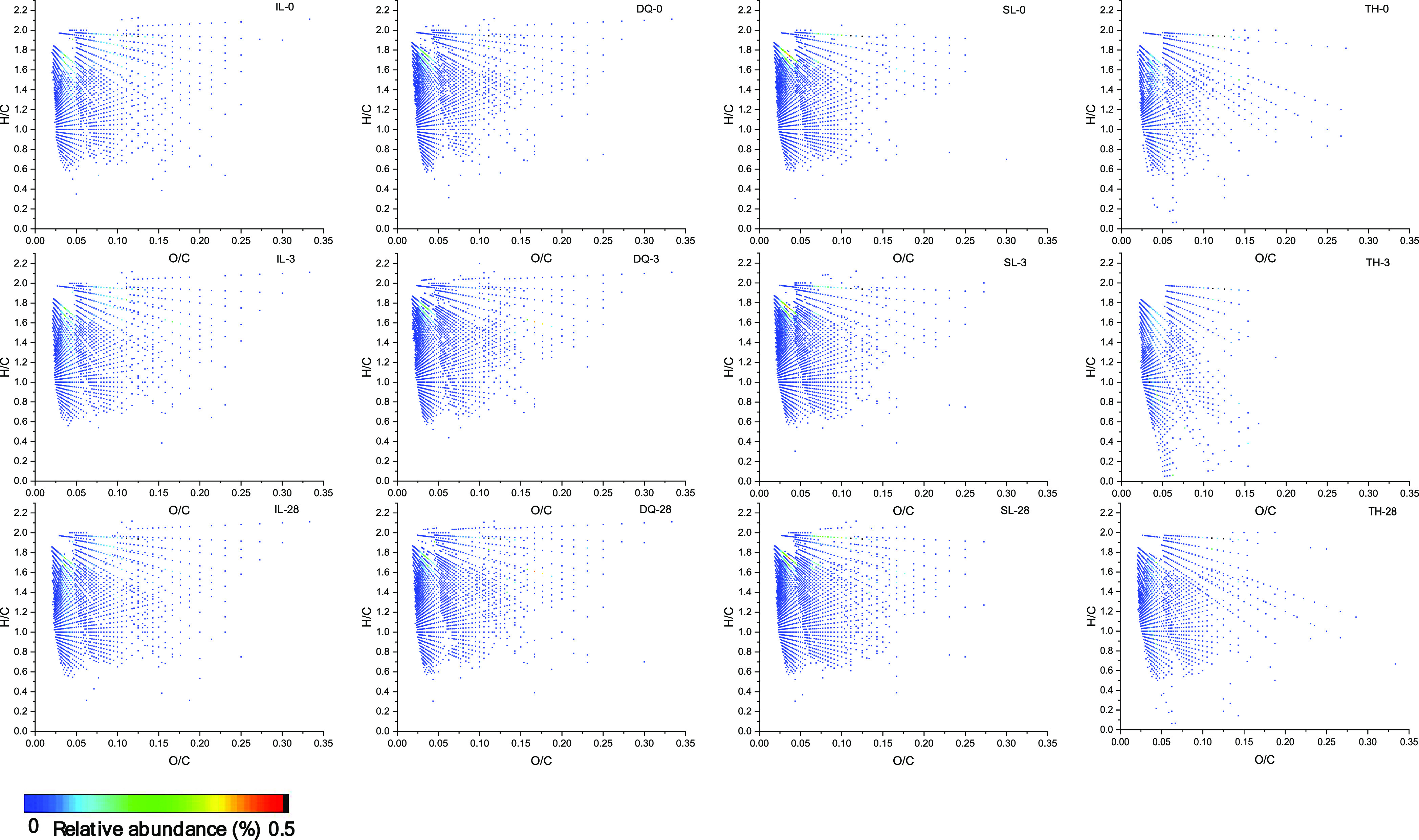
Van Krevelen diagrams for all classes containing oxygen in crude oils during the weathering process.

Van Krevelen diagrams plot the molar ratio of hydrogen to carbon (H/C ratio) versus the molar ratio of oxygen to carbon (O/C ratio), and have been applied extensively to complex oil samples.^37^Figure 10 shows van Krevelen diagrams generated from elemental compositions derived from negative-ion ESI FT-ICR MS for all classes containing oxygen in crude oils during the weathering process. Compared with the four crude oils, the IL crude oil had a relatively higher H/C ratio, indicating the abundant LMW hydrocarbons; DQ and SL crude oils had a relatively higher O/C ratio, indicating the abundant polar compounds. During the weathering process, the decreasing trend of the H/O ratio showed the depletion of LMW *n*-alkanes and PAHs; the increasing trend of the O/C ratio suggested the concurrent oxidation during the volatilization process. As for the SL crude oil, the compounds with an O/C ratio of 0.1–0.15 and a H/C ratio of 0.6–1.0 significantly appeared. As for the DQ crude oil, the compounds with an O/C ratio of 0.15–0.25 and a H/C ratio of 0.5–1.0 gradually appeared. The results showed that there were some compounds that underwent an oxidation transformation process.

## Conclusions

In this work, compositional analyses of *n*-alkanes, PAHs, and heteroatomic compounds in four crude oil samples during the weathering process were performed using GC-FID, GC-MS, and ESI FT-ICR MS. The primary findings are summarized as follows:

(1) The *n*-alkanes are distributed in the range of C8–C39, and the LMW *n*-alkanes in IL and TH crude oils were more abundant, which were distributed in the range of C9–C17. The *n*-alkanes in DQ and SL crude oils were distributed in the range of C15–C30. The low-carbon *n*-alkanes C8–C12 were greatly affected by the weathering process, and the weathering depletion was close to 100%. The weathering loss gradually decreased with the increase in the carbon number.
(2) Naph series compounds were the most abundant PAHs, which had a high alkylation degree, followed by Phen and DBT. PAHs in crude oils were mainly composed of two branched-chain components. The depletion of LMW naphthalene and alkylated homologues was greatly affected by the weathering process. With the increase in the alkylation degree, the weathering resistance was enhanced.
(3) In the negative-ion ESI FT-ICR MS mode, the species of N1, O1, O2, N1S1, N1O1, N2, and O1S1 class compounds were detected in neutral nitrogen compounds and acidic compounds of four crude oils. With the increase in the weathering process, the relative abundances of N1, N2, and NS compounds in the four crude oils decreased, while the relative abundances of NO, NO2, and O3S compounds increased gradually, and the NO and NO2 compounds with different condensation degrees increased significantly to a certain extent. This indicated that in addition to the volatilization of hydrocarbon compounds in the weathering process, nitrogen compounds were also oxidized to a certain extent during the weathering process, which enriched the understanding of the short-term weathering process of petroleum.

## Materials and Methods

### Oil Samples and the Weathering Experiment

The crude oils used in the experiment, i.e., Iranian light (IL) crude oil, Daqing (DQ) crude oil, Shengli (SL) crude oil, and Tahe (TH) crude oil, were provided by SINOPEC Research Institute of Petroleum Processing (Beijing, China). Each 400 mL of crude oil was added to four brown glass bottles, respectively, and the bottles were inflated by an air pump connected with a glass tube. Due to the high content of macromolecular components, DQ and SL crude oils were solidified at room temperature. To ensure the fluidity of a liquid, the weathering experiment was conducted at 55 °C in a water bath. The weathering experiment was carried out for 28 days, and crude oil samples were taken at 0, 3rd, and 28th days, respectively. The mass was measured before and after the weathering process to calculate the mass loss rate. The contents of *n*-alkanes (C9–C40) and PAHs in crude oil samples were analyzed by GC-FID and GC-MS, respectively. The heteroatomic compounds in crude oil samples were analyzed by FT-ICR MS. The boiling-point distribution before and after weathering was measured by ASTM D 6352.

### Petroleum Analyses

For the analyses of *n*-alkanes and PAHs,^38,39^ the crude oil samples were dissolved in *n*-hexane, and passed through a silica gel column with anhydrous sodium sulfate on the top. The fractions of *n*-alkanes and PAHs were eluted with *n*-hexane and *n*-hexane/dichloromethane mixture (v/v = 1:1), respectively.

The *n*-alkanes (C9–C40) were determined on an Agilent 7890B GC-FID system equipped with an HP-1 column (50 m × 200 μm × 0.5 μm). The injector and FID temperatures were 310 and 320 °C, respectively. The air and hydrogen flows were 40 mL/min. The column temperature was initiated at 40 °C (held for 10 min) and increased to 315 °C at 10 °C/min (held for 67 min). Nitrogen was used as a carrier gas with a column flow of 1.0 mL/min. Prior to sample analysis, GC-MS was calibrated with the PAH standard mixture. A five-point calibration curve that demonstrated the linear range of PAH analysis was established. The relative response factors (RRFs) for each target PAHs were calculated relative to the internal standard d14-terphenyl. The PAHs (naphthalene, phenanthrene, dibenzothiophene, chrysene, fluorene, and their alkylated compounds) were analyzed on an Agilent 7890B/5977B GC-MS equipped with a DB-5MS column (30 m × 250 μm × 0.25 μm) and an electron ionization (EI) source (70 eV). Programmed temperature conditions were set as follows: initial 60 °C (held for 2 min), increased to 140 °C at 50 °C/min (held for 10 min), and to 300 °C at 3 °C/min (held for 10 min). Quantitation of target PAHs was performed in a selected ion monitoring (SIM) mode with RRFs for each compound determined during the instrument calibration. The PAH concentrations in samples were within the range of the calibration curve.

For the analysis of heteroatomic compounds, the crude oil samples were dissolved with toluene to produce a 1 mg/mL solution and were further diluted to yield a final concentration of 250 μg/mL in a toluene/methanol (1:1 v/v) solution with a 1% NH_4_OH solution added to enhance the ionization efficiency under a negative-ion ESI mode.^40^ The instrumental analysis was performed on a SolariX FT-ICR MS system (Bruker Company), which was equipped with a magnetic field strength of 15 T, an ESI ionization source, and a negative-ion mode. Through a syringe pump, samples were injected at a flow rate of 180 μL/h into the ESI source. Under the negative-ion ESI mode, a 4.0 kV spray shield voltage, a 4.5 kV capillary column introduced voltage, and a 240 V capillary column end voltage were used. The ions were stored in an argon-filled collision cell for 1 s and then transferred to the ICR cell with 0.8 ms flight time. A total of 128 scans were accumulated and averaged to improve the signal-to-noise ratio of the mass spectrum. The mass range was 200–700 Da, and the size of the data set was set to 4 megawords.

## Supporting Information Available

Gas chromatograms of *n*-alkanes in crude oil samples (Figure S1), gas chromatograms of PAHs in crude oil samples (Figure S2), and ESI FT-ICR MS spectra of crude oil samples (Figure S3) (PDF)
