# Supplementary material for: Molecular Analyses of Petroleum Hydrocarbon Change and Transformation during Petroleum Weathering by Multiple Techniques
Source: ACS Omega. 2021 Aug 31;6(36):23222–32. doi: 10.1021/acsomega.1c02846 (PMC8444223; doi:10.1021/acsomega.1c02846)
Supplement: Supplementary file 1 — ao1c02846_si_001.pdf [file ao1c02846_si_001.pdf]

Supporting Information for

Molecular Analyses of Petroleum Hydrocarbon Change and Transformation during Petroleum Weathering by Multiple-Techniques

Yazhuo Li[a,b], Hui Wang[c], Zhengqing Cai[d], Jibiao Zhang[a,*], Jie Fu[b,*]

[a] *Department of Environmental Science and Engineering, Fudan University, Shanghai 200433, China*

[b] *School of Environmental Science and Engineering, Huazhong University of Science and Technology, Wuhan 430074, China*

[c] *SINOPEC Research Institute of Petroleum Processing, Beijing 100083, China*

[d] *National Engineering Laboratory for High-concentration Refractory Organic Wastewater Treatment Technologies, East China University of Science and Technology, Shanghai 200237, China*

[*] Corresponding authors.

E-mail address: jbzhang@fudan.edu.cn (J. Zhang), jiefu@hust.edu.cn (J. Fu)

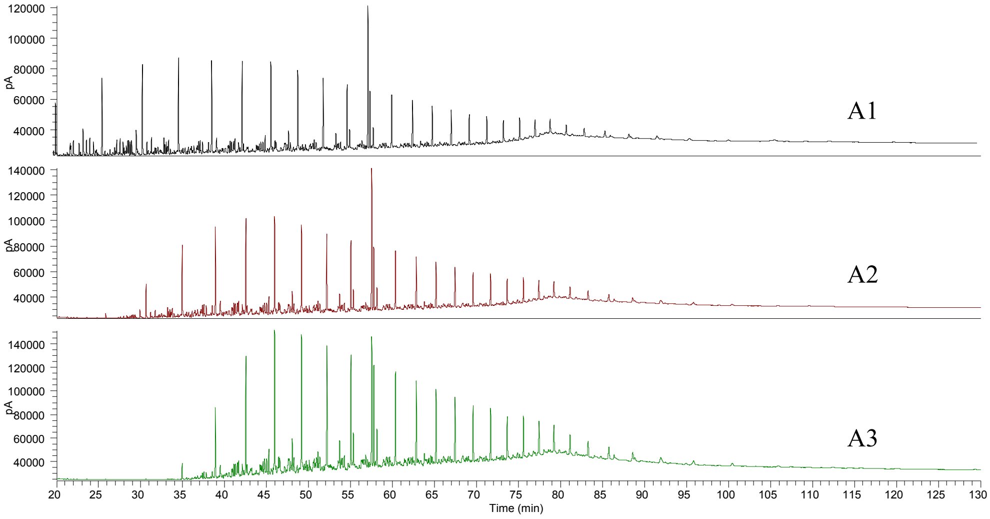
A1
A2
A3
pA
Time (min)

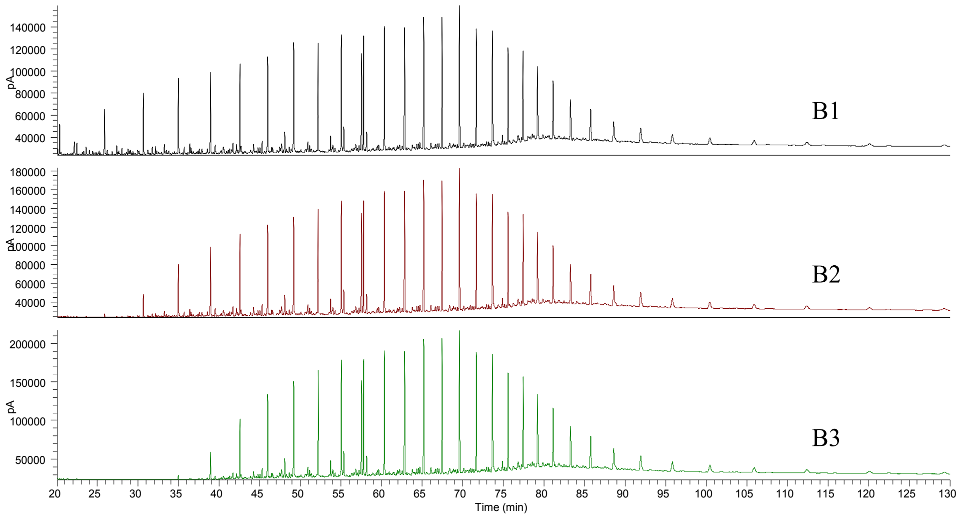
B1
B2
B3
pA
Time (min)

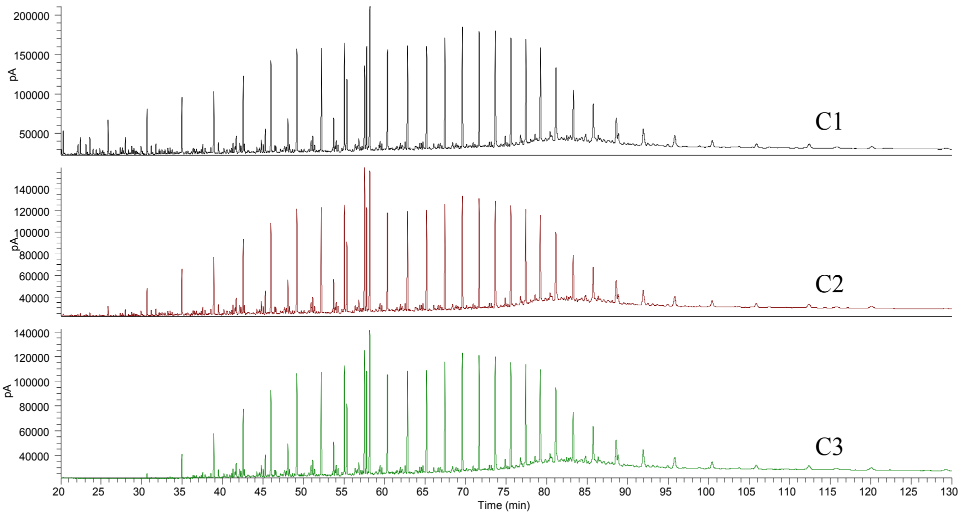
C1
C2
C3
pA
Time (min)

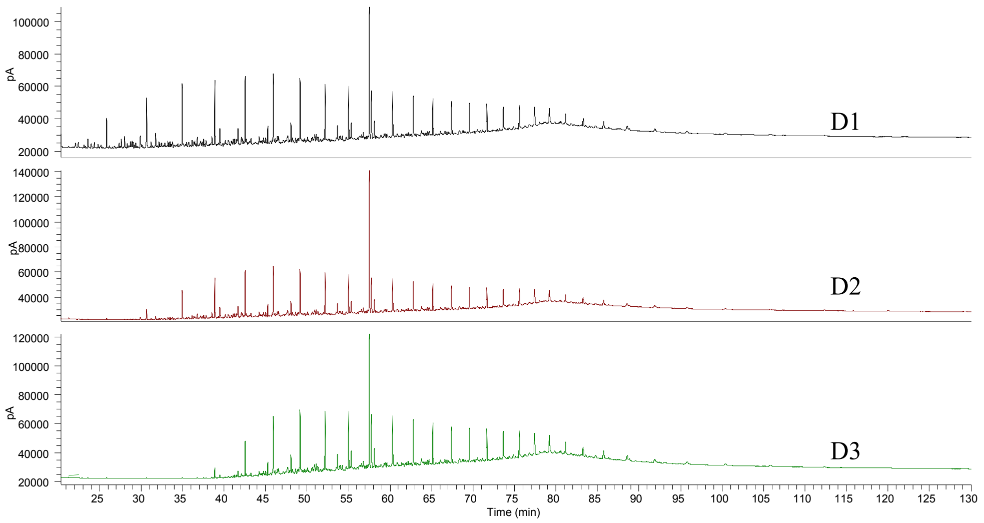

Figure S1. Gas chromatograms of n-alkanes in crude oil samples. A1-A3, IL crude oils of weathering for 0, 3 and 28 days; B1-B3, DQ crude oils of weathering for 0, 3 and 28 days; C1-C3, SL crude oils of weathering for 0, 3 and 28 days; D1-D3, TH crude oils of weathering for 0, 3 and 28 days.

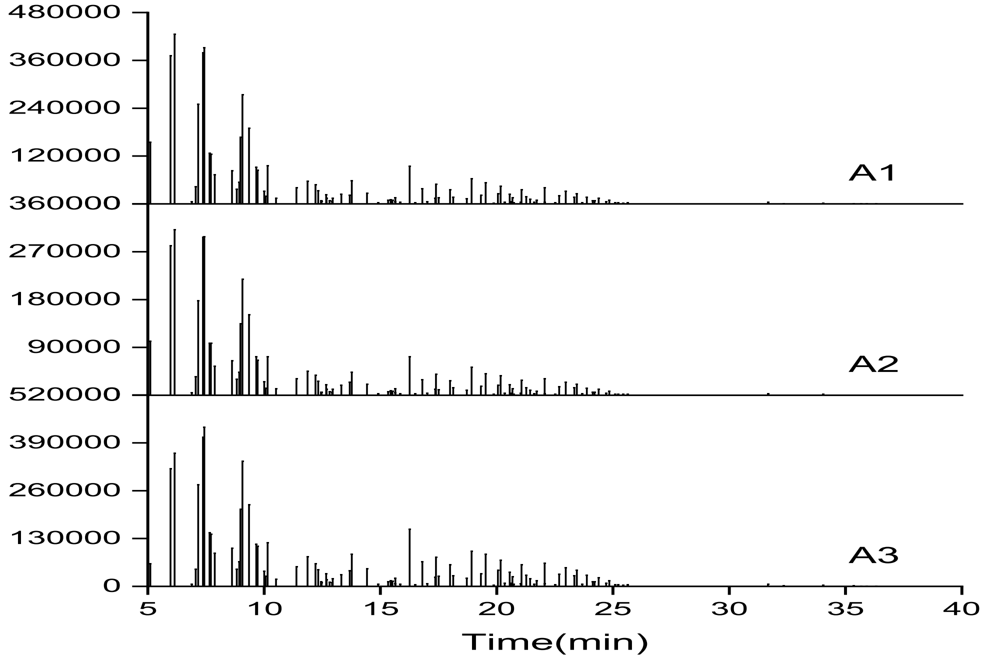
480000
360000
240000
120000
360000
270000
180000
90000
520000
390000
260000
130000
0
A1
A2
A3
5
10
15
20
25
30
35
40
Time(min)

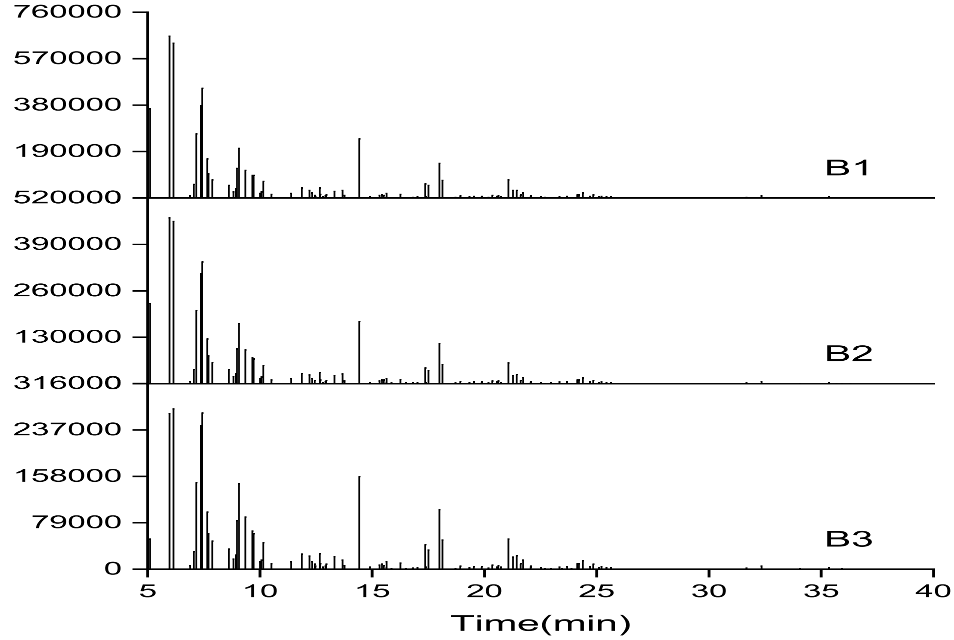
760000
570000
380000
190000
520000
390000
260000
130000
316000
237000
158000
79000
0
B1
B2
B3
5
10
15
20
25
30
35
40
Time(min)

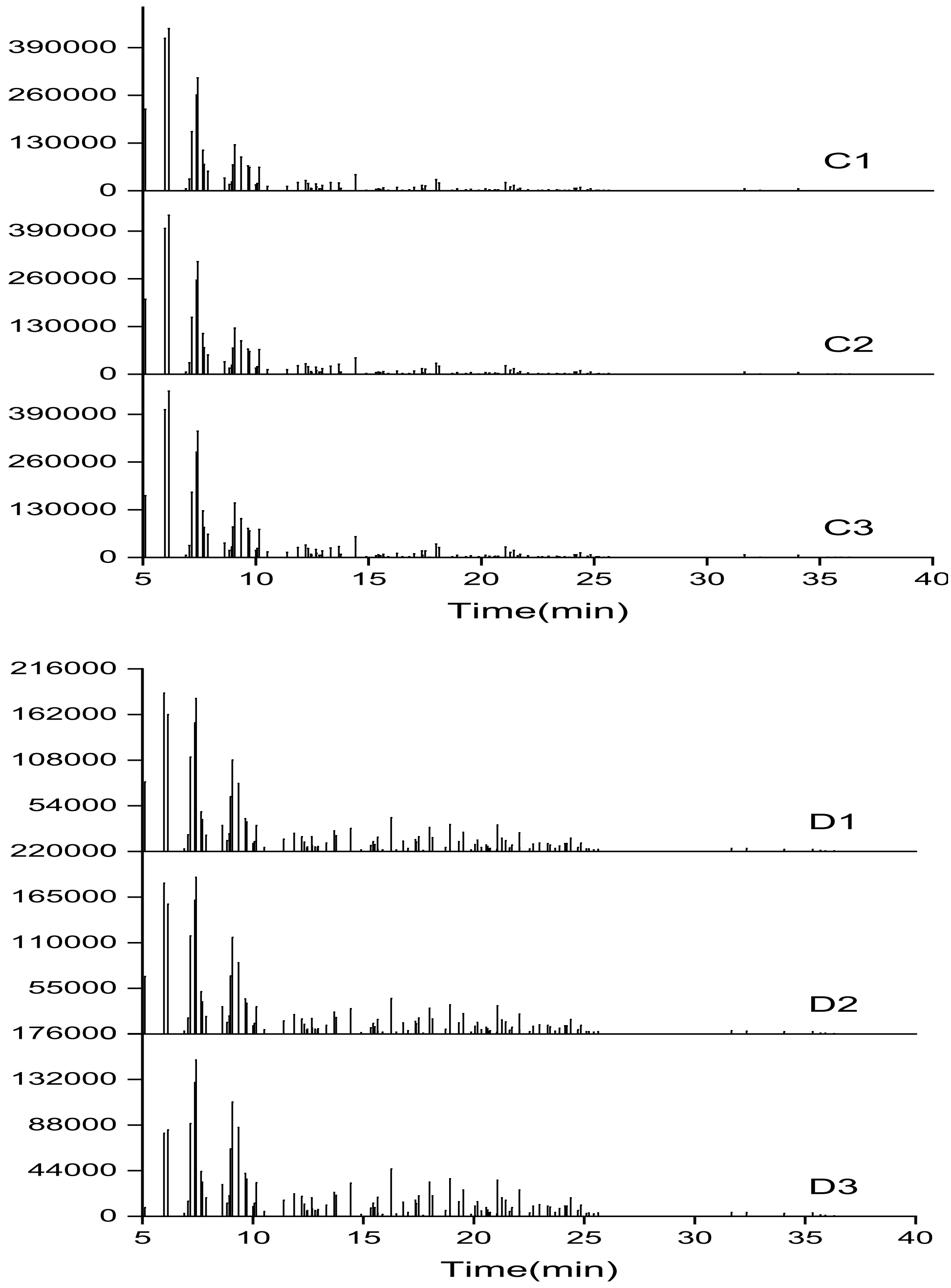

Figure S2. Gas chromatograms of PAHs in crude oil samples. A1-A3, IL crude oils of weathering for 0, 3 and 28 days; B1-B3, DQ crude oils of weathering for 0, 3 and 28 days; C1-C3, SL crude oils of weathering for 0, 3 and 28 days; D1-D3, TH crude oils of weathering for 0, 3 and 28 days.

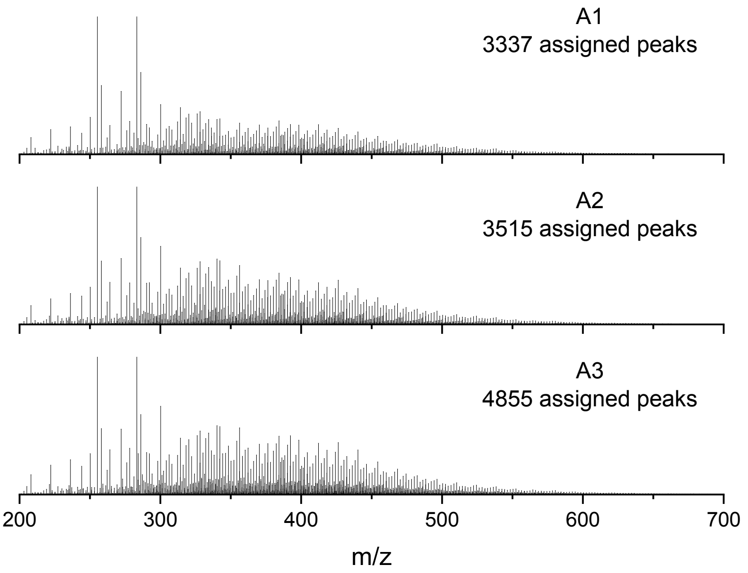
A1
3337 assigned peaks
A2
3515 assigned peaks
A3
4855 assigned peaks
200
300
400
500
600
700
m/z

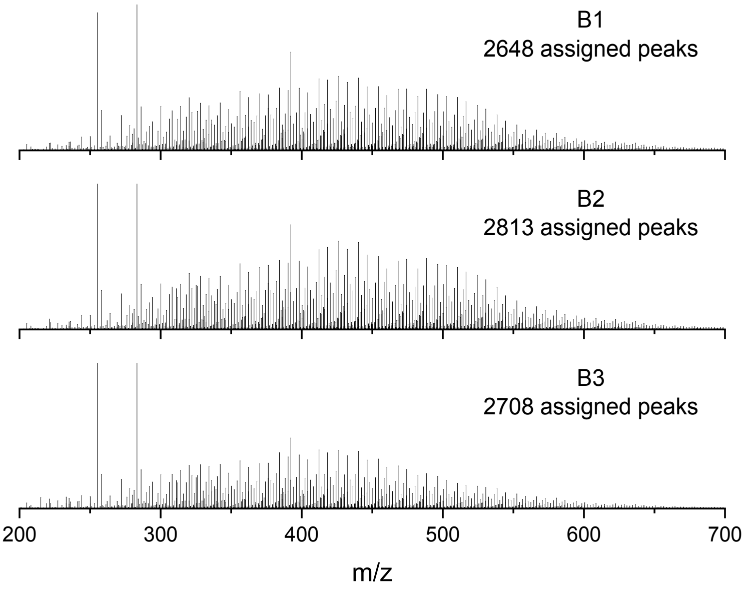
B1
2648 assigned peaks
B2
2813 assigned peaks
B3
2708 assigned peaks
200
300
400
500
600
700
m/z

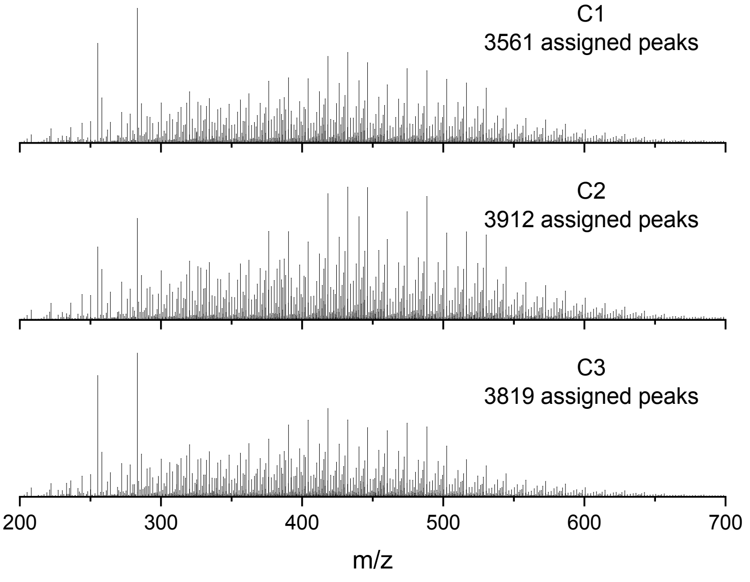
C1
3561 assigned peaks
C2
3912 assigned peaks
C3
3819 assigned peaks
200
300
400
500
600
700
m/z

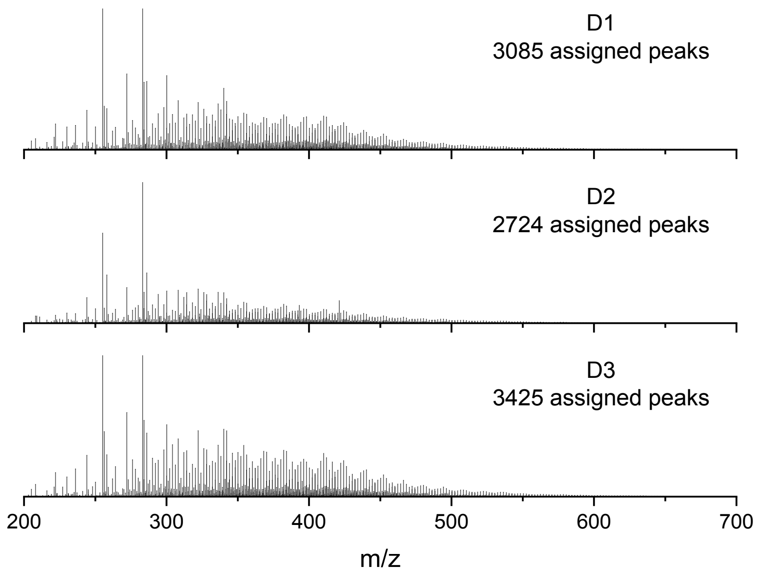

Figure S3. ESI FT-ICR MS spectra of crude oil samples. A1-A3, IL crude oils of weathering for 0, 3 and 28 days; B1-B3, DQ crude oils of weathering for 0, 3 and 28 days; C1-C3, SL crude oils of weathering for 0, 3 and 28 days; D1-D3, TH crude oils of weathering for 0, 3 and 28 days.

Table S1. Boiling point distribution before and after weathering.

Boiling point (℃)	IL		IL-28		DQ		DQ-28		SL		SL-28		TH		TH-28	
	%	∑%	%	∑%	%	∑%	%	∑%	%	∑%	%	∑%	%	∑%	%	∑%
IBP-100	-	-	-	-	0.6	0.6	-	-	-	-	-	-	0.9	0.9	-	-
100-120	-	-	-	-	0.4	1	-	-	-	-	-	-	0.9	1.8	0.8	0.8
120-140	-	-	-	-	0.6	1.6	-	-	0.6	0.6	-	-	1.3	3.1	1.2	2
140-160	-	-	-	-	0.8	2.4	-	-	0.6	1.2	-	-	1.7	4.8	1	3
160-180	-	-	-	-	0.8	3.2	-	-	0.7	1.9	-	-	1.8	6.6	0.8	3.8
180-200	-	-	-	-	0.9	4.1	-	-	0.9	2.8	0.8	0.8	2	8.6	0.8	4.6
200-220	1.6	1.6	-	-	1	5.1	0.6	0.6	1.2	4	1.2	2	2.3	10.9	1.6	6.2
220-240	2.2	3.8	1.2	1.2	1	6.1	0.9	1.5	1.2	5.2	1.7	3.7	2.5	13.4	1.2	7.4
240-260	3.1	6.9	1.7	2.9	1.3	7.4	1.5	3	1.6	6.8	2.1	5.8	2.8	16.2	2.4	9.8
260-280	3.3	10.2	2.1	5	1.4	8.8	1.7	4.7	1.6	8.4	2.2	8	2.8	19	2.6	12.4
280-300	3.9	14.1	2.6	7.6	1.9	10.7	2.3	7	1.9	10.3	3.4	11.4	3.3	22.3	3	15.4
300-320	3.7	17.8	2.8	10.4	1.8	12.5	2	9	2	12.3	3.3	14.7	3	25.3	3.2	18.6
320-340	3.6	21.4	2.9	13.3	1.8	14.3	2.3	11.3	2	14.3	2.8	17.5	3.1	28.4	2.2	20.8
340-360	3.5	24.9	3	16.3	1.9	16.2	2.2	13.5	2.2	16.5	2.5	20	2.9	31.3	2.6	23.4
360-380	3.5	28.4	3	19.3	2.1	18.3	2.5	16	2.2	18.7	2.9	22.9	2.8	34.1	2.2	25.6
380-400	3.4	31.8	3.2	22.5	2.2	20.5	2.5	18.5	2.3	21	3	25.9	2.9	37	2.8	28.4
400-420	3.5	35.3	3.5	26	2.5	23	2.9	21.4	2.5	23.5	3.7	29.6	2.9	39.9	3	31.4
420-440	3.7	39	3.6	29.6	2.9	25.9	3.2	24.6	2.5	26	4.9	34.5	3	42.9	5	36.4
440-460	3.3	42.3	3.3	32.9	2.7	28.6	3	27.6	2.4	28.4	4.2	38.7	2.8	45.7	4	40.4
460-480	3.2	45.5	3.2	36.1	2.6	31.2	2.9	30.5	2.3	30.7	3.2	41.9	2.7	48.4	3.4	43.8
480-500	3.3	48.8	3.3	39.4	2.8	34	2.9	33.4	2.3	33	3.4	45.3	2.7	51.1	3.4	47.2
500-520	3.2	52	3.5	42.9	2.9	36.9	3.1	36.5	2.5	35.5	3.3	48.6	2.6	53.7	3.4	50.6
520-540	3.4	55.4	3.6	46.5	3.3	40.2	3.3	39.8	2.6	38.1	3.4	52	2.8	56.5	3.6	54.2
540-560	3.2	58.6	3.4	49.9	3.2	43.4	3.2	43	2.5	40.6	3.2	55.2	2.6	59.1	3.4	57.6
560-580	3.1	61.7	3.2	53.1	3.2	46.6	3.3	46.3	2.2	42.8	3.1	58.3	2.5	61.6	3.8	61.4
580-600	2.8	64.5	2.9	56	3.1	49.7	3.2	49.5	2.1	44.9	2.9	61.2	2.2	63.8	3.6	65
600-620	2.6	67.1	2.7	58.7	3.1	52.8	3.3	52.8	2	46.9	2.9	64.1	2.2	66	3.4	68.4
620-640	2.4	69.5	2.3	61	3	55.8	3.2	56	1.7	48.6	2.7	66.8	1.9	67.9	2.2	70.6
640-660	2.2	71.7	2.1	63.1	3	58.8	3.2	59.2	1.6	50.2	2.5	69.3	1.8	69.7	3	73.6
660-680	2	73.7	1.9	65	3	61.8	3.1	62.3	1.4	51.6	2.4	71.7	1.7	71.4	2.6	76.2
680-700	1.8	75.5	1.6	66.6	2.9	64.7	3.2	65.5	1.3	52.9	2.3	74	1.4	72.8	2	78.2
700-720	1.6	77.1	1.3	67.9	2.9	67.6	3.1	68.6	1	53.9	1.9	75.9	1.3	74.1	2	80.2
720-750	1.6	78.7	1.1	69	3.2	70.8	3.3	71.9	0.9	54.8	2	77.9	1.2	75.3	1.2	81.4

IBP: initial boiling point
